# PPIntegrator: semantic integrative system for protein–protein interaction and application for host–pathogen datasets

**DOI:** 10.1093/bioadv/vbad067

**Published:** 2023-06-01

**Authors:** Yasmmin Côrtes Martins, Artur Ziviani, Maiana de Oliveira Cerqueira e Costa, Maria Cláudia Reis Cavalcanti, Marisa Fabiana Nicolás, Ana Tereza Ribeiro de Vasconcelos

**Affiliations:** Bioinformatics Laboratory, National Laboratory for Scientific Computing, Petrópolis 25651-076, Brazil; Data Extreme Laboratory (DEXL), National Laboratory for Scientific Computing, Petrópolis 25651-076, Brazil; Bioinformatics Laboratory, National Laboratory for Scientific Computing, Petrópolis 25651-076, Brazil; Systems and Computation, Military Institute of Engineering, Rio de Janeiro 22290-270, Brazil; Bioinformatics Laboratory, National Laboratory for Scientific Computing, Petrópolis 25651-076, Brazil; Bioinformatics Laboratory, National Laboratory for Scientific Computing, Petrópolis 25651-076, Brazil

## Abstract

**Summary:**

Semantic web standards have shown importance in the last 20 years in promoting data formalization and interlinking between the existing knowledge graphs. In this context, several ontologies and data integration initiatives have emerged in recent years for the biological area, such as the broadly used Gene Ontology that contains metadata to annotate gene function and subcellular location. Another important subject in the biological area is protein–protein interactions (PPIs) which have applications like protein function inference. Current PPI databases have heterogeneous exportation methods that challenge their integration and analysis. Presently, several initiatives of ontologies covering some concepts of the PPI domain are available to promote interoperability across datasets. However, the efforts to stimulate guidelines for automatic semantic data integration and analysis for PPIs in these datasets are limited. Here, we present PPIntegrator, a system that semantically describes data related to protein interactions. We also introduce an enrichment pipeline to generate, predict and validate new potential host–pathogen datasets by transitivity analysis. PPIntegrator contains a data preparation module to organize data from three reference databases and a triplification and data fusion module to describe the provenance information and results. This work provides an overview of the PPIntegrator system applied to integrate and compare host–pathogen PPI datasets from four bacterial species using our proposed transitivity analysis pipeline. We also demonstrated some critical queries to analyze this kind of data and highlight the importance and usage of the semantic data generated by our system.

**Availability and implementation:**

https://github.com/YasCoMa/ppintegrator, https://github.com/YasCoMa/ppi_validation_process and https://github.com/YasCoMa/predprin.

## 1 Introduction

Semantic web technologies such as ontologies, linked data and query drivers have been applied in many bioinformatics applications since the number of datasets representing the biological area has tremendously increased in the last two decades (Hoehndorf *et al.*, 2015). The data in the semantic web are represented as triples, which are composed of subject, predicate and object following the Resource Description Framework (RDF) pattern and the Linked Open Data principles (Bizer *et al.*, 2009). Several ontologies implemented in RDF emerged to model the concepts and represent the vast biological area; the most known case is the Gene Ontology (GO), a knowledgebase source of information on the functions of genes (Ashburner *et al.*, 2000). The GO contains a set of terms in its vocabulary concerning three main topics, which are cellular component, molecular function and biological process. Many datasets available in the semantic web come from data stored in relational databases and undergo a triplification process to be transformed into RDF triples. The Bio2RDF initiative (Callahan *et al.*, 2013) performed a broader semantic annotation of several datasets through several ontologies. Moreover, the NCBO Bioportal (Whetzel *et al.*, 2011) concentrates on many ontologies and has tools to annotate and map ontologies. An update of Bioportal also provides recommendations for biomedical vocabulary topics for dataset annotation (Martínez-Romero *et al.*, 2017). A review of diverse biological semantic data integration initiatives is described in Sima *et al.* (2019).

The protein–protein interactions (PPIs) subarea, crucial for biological research, is not currently covered by the previously mentioned initiatives. The PPIs are physical or functional relations between proteins, essential in all biological processes in living organisms. Consequently, the study of PPIs can reveal the functional mechanisms of any cells (Dhanapalan and Chen, 2007). Due to the importance of these interactions, several public PPI databases have emerged, such as STRING (Szklarczyk *et al.*, 2019), HINT (Das and Yu, 2012) and HPIDB (Ammari *et al.*, 2016). STRING uses text mining, gene neighborhood, gene fusion, co-occurrence, co-expression and experimental validation, as the main detection methods. HINT represents and aggregates PPI data from several validated and literature-curated databases and provides high-quality interaction data for twelve organisms. HPIDB also exports validated and curated PPIs as HINT, but its PPIs belong to the host–pathogen context. These databases only offer the data concerning the predictions and the detection method associated with a score. In this regard, our recently published PredPrIn workflow (Martins *et al.*, 2021) generates data from provenance data related to datasets and experiments, including predictions organized by biological evidence, functional annotations or domain–domain interactions.

Ontologies and controlled vocabularies propose to tackle the semantic annotation of protein interaction (PPI) datasets, as applied in Biomanta (Newman *et al.*, 2008) and Biopax (Demir *et al.*, 2010). Recently, we proposed the OntoPPI ontology to expand the classes and properties of the available PPI ontologies (Martins *et al.*, 2019). Our initiative covers the details and complexity of the types of methods that generate the PPI scores and the organization of the datasets, including the data provided by the PredPrIn workflow.

In recent literature, the efforts for automatic integration and enrichment of PPIs data are directed to specific validated databases, not providing cross-reference databases to correlate protein interactions and predictions. Currently, the LinkedPPI system is the closest to an integration project, which includes Biogrid and several annotation databases, to transform, annotate, and create a knowledgebase to help generate new candidate pairs from curated data sources. However, this method is not flexible with computationally inferred PPIs databases, and the user cannot enrich its own protein interactions dataset, previously predicted by another tool. Although other initiatives (Arzt *et al.*, 2011; Cannataro *et al.*, 2010; Dhanapalan and Chen, 2007; Kazemzadeh *et al.*, 2014) propose annotations and query examples of analysis of PPIs dataset, they do not offer an automatic and efficient way to organize multiple datasets annotation. Also, they are not designed to integrate computationally predicted interactions.

Another essential issue not under consideration by the published strategies is the integration of all the processes of PPI augmentation, prediction and analysis for host–pathogen protein interactions (Basit *et al.*, 2018). Some methods focus only on expanding or predicting PPIs, not on both. Recent work proposed a method to precisely predict host–pathogen PPIs using primary sequence embedding with physicochemical features (Macho Rendón *et al.*, 2022). The prediction consists of the interactome derived from a consensus of a few models. This method has a high computational cost to compute pairwise embedding similarities. Another approach for host–pathogen prediction uses a series of filters such as sequence alignment, domain–domain interaction and biological context or functional annotation (Huo *et al.*, 2015). A similar method for plant-pathogen PPIs prediction also extracts features from sequence comparison and domain–domain interactions, but also structural features such as TM-Score (Zhang and Skolnick, 2005), RMSD (Root Mean Square Distance) among interacting residues, and fraction and several residue contacts preserved in the interaction models (Zheng *et al.*, 2021). All these methods do not provide strategies to generate new potential PPIs for a desired organism. Huo *et al.* (2015) and Zhang and Skolnick (2005) proposed a method to predict host–pathogen interaction that cannot be transferred to another species other than the originally implemented pathogen, *Mycobacterium tuberculosis* and *M.oryzae*. These two methods are not available to test and have no documentation. The recently published HPIPred method (Macho Rendón *et al.*, 2022) is available, is documented with instructions, and can be used with other pathogens. However, it does not recommend new potential PPIs or allow multiple dataset execution or parallelism.

Here, we updated and extended OntoPPI ontology to model protein interactions from one or more species, including support for the association with published articles validating the PPIs. We also introduced a novel host–pathogen PPIs discovery process to enrich known validated datasets through a transitivity analysis. We performed the PPI augmentation, coupled with an exhaustively evaluated PredPrIn pipeline, to predict PPIs. We also offer an evaluation tool to guide the users to correlate relevant articles to the PPIs (Martins *et al.*, 2021). Finally, we designed the PPIntegrator method to describe the information of any dataset of protein interactions using all possible concepts of OntoPPI to enrich this information for the semantic context. This method contains a structure to standardize this type of biological data, which has five steps: (i) Generate annotations about the experiments and provenance of the datasets with interactions as well as the organisms involved; (ii) Generate the knowledge base about proteins with their functional annotations; (iii) Generate the descriptions about the scores, interactions, detection methods used for the score and possible links to scientific literature articles; (iv) Data fusion the datasets items (interactions) organized by organism; (v) Exportation for AllegroGraph tool to make analyses and queries on the data (https://allegrograph.com/). We aim to contribute by showing a strategy to triplify, integrate with other datasets and ontologies offering the interlinking, and apply mapping among interactions from different PPI datasets for the organisms. We have built all the steps of PPIntegrator to comply with FAIR data principles (Wilkinson *et al.*, 2016).

## 2 Methodology

The background concepts about the semantic web are in the Supplementary Material S1. This section presents the properties added to OntoPPI (Martins *et al.*, 2019) ontology (more details of OntoPPI are in Supplementary Material S2) to represent other crucial information of PPI datasets. It complements evidence supporting the interaction in the scientific literature. We also explain the steps of the PPIntegrator strategy: (i) dataset preparation; (ii) triplification process (experiment configuration, protein features annotation and score results for each detection method), and Data fusion between databases. We applied this process to predict novel potential host–pathogen PPIs by a transitivity analysis.

### 2.1 Update of the OntoPPI ontology

**Figure 1.**
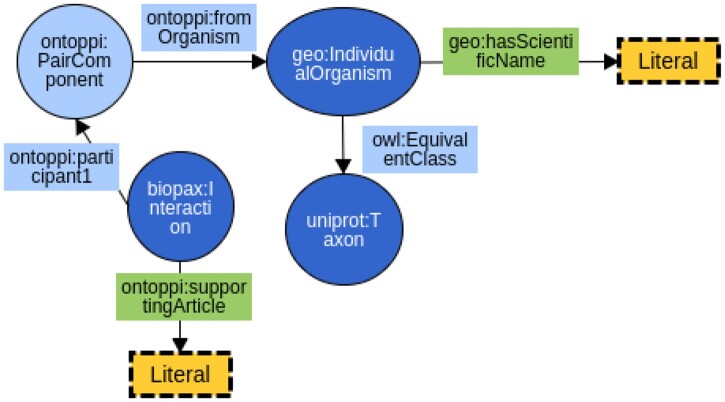
Update of the former OntoPPI ontology. Ellipses (starting by ontoppi:) represent OntoPPI classes, the other ellipses (starting by geo, biopax and uniprot) mean external classes used on OntoPPI (belonging to Other linked vocabularies), object properties are identified by the rectangles ontoppi:fromOrganism and owl:EquivalentClass, data properties are the rectangles without border, and dashed rectangles mean the literal values that data properties may have. We added the datatype property to support evidence found in published literature, linking an interaction to the Pubmed identifiers. Also, we addressed the specification of the protein organism by adding the object property fromOrganism connecting to an instance of the Taxonomy class in the Uniprot ontology (http://purl.uniprot.org/core), reusing the class *IndividualOrganism* and the property *hasScientificName*

Although the former OntoPPI covers all the main concepts for the domain of protein interaction experiments, we updated it here with metadata concerning scientific publications and the organism to which proteins belong (as shown in Figure 1). We imported the class *IndividualOrganism* from GeoSpecies Ontology (https://bioportal.bioontology.org/ontologies/GEOSPECIES/?p=summary) (one of the ontologies hosted in the BioPortal), and the newly added property *fromOrganism* links the interaction participant *ontoppi: PairComponent* (we refer to it with the namespace geo) to the organism instance. Besides, from the schema ontology of Uniprot (http://purl.uniprot.org/core), organized into 178 classes, we link the class *Taxon* (corresponding to the taxonomy of organisms in the database) to the corresponding instance in the GeoSpecies to describe it in a subsequent task. We added the axiom expressing the equivalence between *uniprot: Taxon* and *geo: IndividualOrganism*, using the property *owl: EquivalentClass*. This information is crucial when the interactions come from different organisms and hence filters the semantic data when applying a deeper analysis and cross-species comparison.

This modification allows for the annotation of PPI datasets in a set of contexts, using PPIs derived only from one organism such as Fly or modeling host–pathogen protein interactions, in which the proteins belong to distinct species such as Humans and Bacteria.

The other property is *ontoppi: supportingArticle*, and its domain is an instance of *biopax: Interaction*, which means that, for some reference datasets like HINT, the interaction has already been validated or described by a scientific paper. Besides the values belonging to type Literal (string values), we used a pattern to add the papers for the interaction results. The values assigned to this new property may be identifiers of a public scientific literature database known as PubMed (https://www.ncbi.nlm.nih.gov/pubmed/). This database is divided into two sections: Pubmed and Pubmed central. The first one is for the papers that may not have all the content available (just the title and abstract sometimes), and the second one has the full paper content always available. The exported tables indicate the articles with full-text findings using the *pmc* identifier and those containing just abstracts using *pubmed*, aside from the numerical paper identifier.

### 2.2 Host–pathogen PPIs discovery process

**Figure 2.**
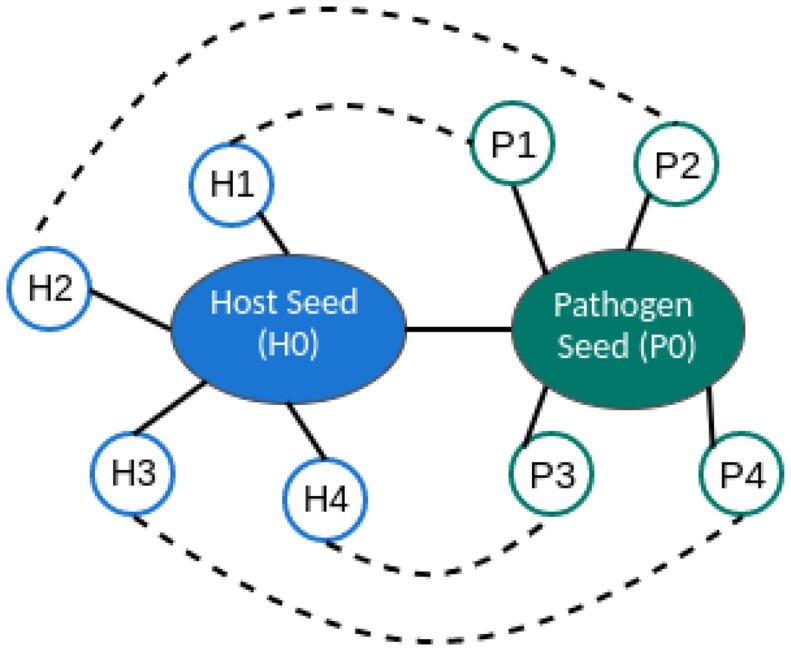
Transitivity analysis in PPI network. The big left circle is the host protein seed, and the small circles are the host seed partners. The big right circle is the pathogen seed with its respective partners (in small right circles). The continual edges represent validated interactions (e.g. H0-P0, P0-P2, H0-H3) retrieved from the databases HINT and HPIDB, and the dashed edges represent the candidate links among partners of the host and pathogen seeds. The candidate generation considers all possible combinations (e.g. P1-H1, P2-H4, P1-H3, P3-H1), but only some candidate links were represented to keep the image clear

The host–pathogen PPIs discovery process (available at: https://github.com/YasCoMa/hppidiscovery.git) aims to predict and validate through published literature the potential new interactions between pathogen and host. The process takes as input a dataset of known validated host–pathogen PPIs, such as those available in HPIDB. The process handles each organism separately and enriches the original host–pathogen datasets by a transitivity analysis. The rationale behind our transitivity analysis is similar to those used in the Bidirectional Best Hits (BBHs) ortholog group construction (Fang *et al.*, 2010). The main idea of transitivity is that we have a known validated interaction with a host and a pathogen protein seed. The new candidates are formed by combining the direct partners of each of these seeds (illustrated in Figure 2).

**Figure 3.**
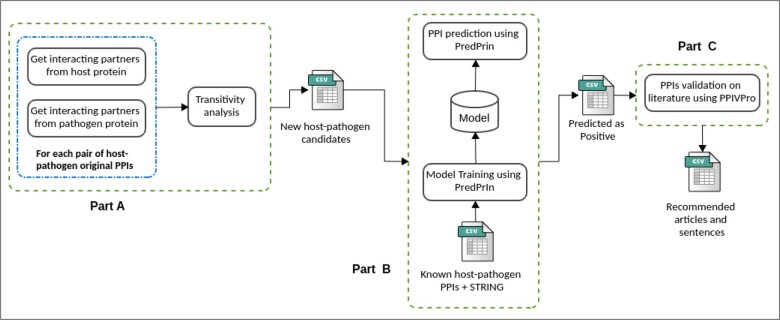
Overall workflow for host–pathogen discovery. Part (A) refers to the PPI augmentation process to generate new potential PPIs from the seeds in the HPIDB-validated datasets using the transitivity analysis (enrichment from the seeds’ partners). Part (B) is responsible for training the PredPrin model using the validated PPIs. The trained model is then applied to predict the new PPIs candidates. The positively predicted PPIs will be post-processed (Part C) to discover specific articles with more information about these interactions

The host–pathogen PPIs discovery workflow (Figure 3) begins (part A) by extracting the non-redundant proteins contained in the known PPIs dataset, organized by the organism. This set of proteins is used as seeds to search for high-confidence neighbors from the STRING PPI network (using a cutoff of 900) and then combined with the host proteins, maintaining the original seed to which it was associated. We use the shortest paths with only one edge of distance to retrieve the direct neighbors of the seed proteins from the known protein interactions to generate the new PPI candidates. This step also removes the possible overlaps (repeated interactions) compared to the original validated dataset (HPIDB). The potential new PPIs representing the test set (to be predicted by the PredPrIn) are then evaluated (part B) by an Adaboost classifier (Schapire, 2013) trained model built from all validated datasets of host–pathogen PPIs. The predicted probabilities of each data point for the target class were provided to estimate the performance metrics and to generate the Precision-Recall and Receiver Operating Characteristic (ROC) curves. The recently published PredPrIn method executes training and prediction tasks (Martins *et al.*, 2021). Finally, the positively predicted interactions are applied to the PPIVPro (available at: https://github.com/YasCoMa/ppi_validation_process) pipeline (part C) to retrieve the articles that may corroborate or provide more information about them. This validation process recovers, through text mining of scientific articles published on PubMed, the sentences where a pair of proteins are included in an interaction.

### 2.3 PPIntegrator

**Figure 4.**
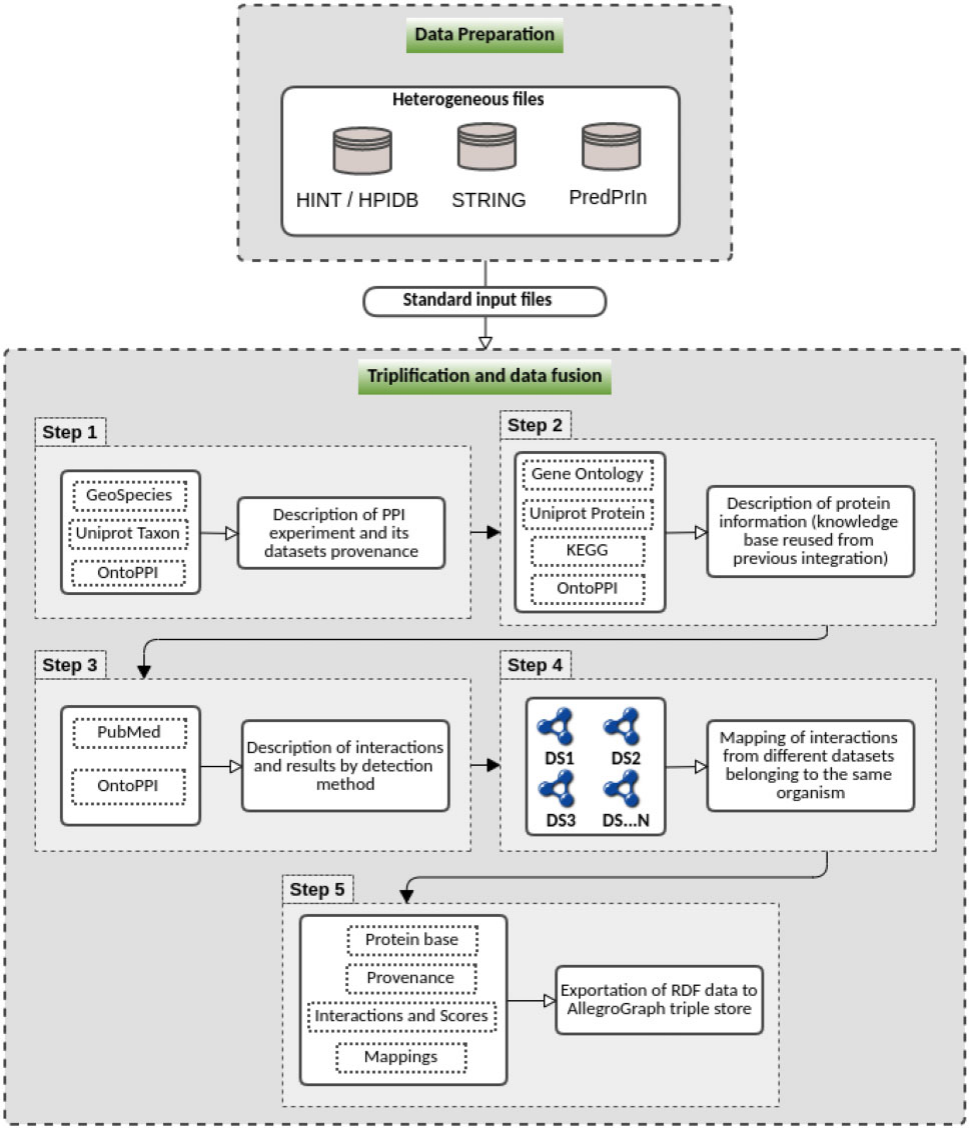
PPIntegrator pipeline with data preparation, triplification modules, and the steps described inside the modules. The data preparation module formates the heterogeneous data recovered from databases to generate standard input data for the subsequent module. The triplification and data fusion module handles the identification of organisms of each protein (Step 1), generates the annotations for the experiment provenance (Step 1), functional annotations of proteins (Step 2), description of the PPI predictions according to the detection methods (Step 3), and mapping the interactions of overlapping protein pairs with datasets (DS1, DS2, DS3, …, DSN) for data fusion (Step 4). This module also has the option to export to the triple-store database AllegroGraph (Step 5)

PPIntegrator integrates protein interaction data using semantic web standards. It automatically describes the PPIs prediction data into triples according to the RDF—Resource Description Framework (Bizer *et al.*, 2009) and interlinks this data to other databases, such as UniProt and KEGG, using their SPARQL (https://www.w3.org/TR/sparql11-query/) (SPARQL Protocol and RDF Query Language) endpoints. Two major modules compose our integration system (Figure 4), namely: (i) data preparation and (ii) triplification and data fusion.

#### 2.3.1 Data preparation module

The data preparation module aims to process the data to input the triplification and data fusion module (first module of Figure 4). We built an algorithm to prepare, extract and transform data from the protein interaction references databases (STRING, HINT and host–pathogen interactions HPIDB). Moreover, we use all the results of the workflow (PredPrIn), which exports data of all the steps of PPIs prediction experiments (Martins *et al.*, 2021). The preparation module accepts inputs from four selected databases—HINT, HPIDB, STRING and the data exported by the PredPrIn workflow.

Finally, the PredPrIn workflow exports the data of protein information and the results in scores for each evidence method. This data preparation module aims at generating standard files that will be the input to the next module. The details about the transformation process according to each PPI database are found in Supplementary Material S3.

#### 2.3.2 Triplification and data fusion module

The triplification and data fusion module generate triples for the whole experiment information (second module of Figure 4). The central part of the module was annotated with OntoPPI metadata (see details in Supplementary Material S2).

The experiment information and the datasets with their respective details are annotated using only the classes and properties described in OntoPPI (Step 1, Figure 4). Each PairComponent instance (the protein involved in the interactions) is associated with the organism to which the interactions belong using the GeoSpecies and UniProt Taxon ontologies. We added the semantic descriptions concerning the organism as a query for the UniProt database to obtain the correct taxonomic classification for each protein.

Step 2 annotates protein information from GO ontologies (cellular component, molecular function and biological process), EC (Enzyme commission number) that is a classification system based on the reactions catalyzed by the enzymes, which is retrieved from the KEGG (Kanehisa *et al.*, 2017), and protein family identifiers from the Pfam database (El-Gebali *et al.*, 2019). This step stores a local knowledge graph for reuse later in the triplification processes (Step 2 of Figure 4).

Then, in Step 3, we describe the PPIs and their corresponding proteins and organisms and the prediction score of each evidence method described in the input. We also enrich the graph with Pubmed links when the literature is available (Step 3, Figure 4). To complete the overall description of the organism, we use the owl: sameAs property. So the descriptions of this organism are automatically inferred from the distinct datasets. In this context, we also use this scientific name to declare an instance of *geo: IndividualOrganism* [from taxonomy vocabulary found in Bioportal (https://bioportal.bioontology.org/ontologies)]. We use the *owl: sameAs* property to link to the matching source found in the previous step. Also, we applied the new property added to OntoPPI, which is *ontoppi: fromOrganism* to link the PPI dataset to the organism. The Supplementary Material S4 explains and illustrates the edges and nodes added in the semantic graph in each of these three steps of the PPIntegrator.

Step 4 executes the datafusion by mapping the interaction resources among the PPI datasets with the same pairs of UniProt protein identifiers (Step 4, Figure 4).

Finally, Step 5 exports all the RDF triples files already generated for storing with AllegroGraph. This triple store tool has optimized functions to use embedded code or a graphical interface and allows the storage of millions of triples freely.

## 3 Results and discussion

We applied the host–pathogen PPIs discovery process to enrich the PPI networks of four bacteria species: *Escherichia coli (ecoli), Staphylococcus aureus (aureus), M.tuberculosis (tuber)* and *Pseudomonas aeruginosa (paeru)*. Since there are a few validated PPIs in HPIDB for these species, we expanded the PPI networks using our proposed transitivity analysis. Then, the PPIntegrator was used to integrate the host–pathogen PPIs for these four bacteria species semantically. Thus, the PPIntegrator provides a robust host–pathogen network, including the published literature on PPIs. This analysis receives the host–pathogen datasets of each bacteria as input, gets the seeds from each organism from the interaction, enriches these PPI networks (by transitivity analysis), and then integrates them in the PPIntegrator.

### 3.1 Enrichment of host–pathogen PPIs

We retrieved known experimentally determined host–pathogen interactions for these bacteria from the host–pathogen Interactions Database (HPIDB). These recovered proteins from HPIDB were cross-linked to UniProt entries and used as the positive dataset to build a model for PPI prediction by the PredPrIn tool. The negative dataset was built by extracting all the available proteomes for these species in UniProt (UniProt Consortium, 2019) and randomly generating new candidate PPIs from human and bacterial proteins without overlapping with those from the positive dataset. Both positive and negative datasets contain the same number of PPIs (21 239).

**Figure 5.**
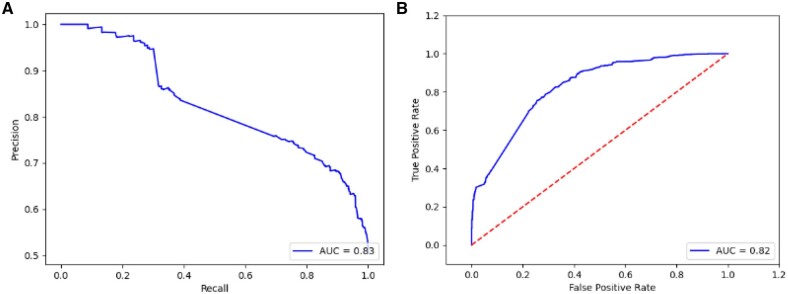
Plots of the model evaluation metrics concerning the precision-recall curve (**A**) and ROC curve (**B**). The dashed straight line depicts the frontier where the classifier has 0.5 of AUC, which is a guideline to evaluate the model, where the reliability of the model is inversely proportional to the distance from the AUC (continuous line)

The model accuracy and precision metric values were approximately 75% with a 10-fold cross-validation strategy. As illustrated in Figure 5, the models in each cross-validation round obtained an area under precision-recall and receiver operating characteristic (ROC) curves of 83% and 82%, respectively. These results show that the model maintains a stable performance. We tested its predictability in the host–pathogen interaction context and showed the trained model with competitive performance. It is relevant to mention that previously the PredPrIn method was exhaustively tested in protein interactions from several organisms (host–host interactions) and obtained optimal evaluation metric values for most of them (Martins *et al.*, 2021). The learning strategy used by PredPrIn uses semantic similarity among gene ontology terms and pathway co-occurrence and they can be transferred to the host–pathogen context. The semantic similarity among the GO terms can still provide relevant components for the classifier because many pathogen proteins have shared or closer annotations in the gene ontology hierarchy. Concerning the pathways co-occurrence, this component can also provide measures to help the classifier because we use the KEGG Ortholog identifiers (KOs) from the protein pair. Using the KOs terms, we retrieve shared pathways of functionally similar proteins. Thus, it is possible to apply the learning strategy tested in host-host interactions for the host–pathogen ones.

The dataset of each bacterial species had interactions from more than one host, so we filtered to get only the ones with human proteins. The gold-standard dataset filtered for human proteins as host decreased few PPIs (Table 1) from all species except for *S.aureus*, of which most PPIs contained a *Bos taurus* protein as host with more interactions. The enrichment strategy (transitivity analysis, Figure 3) used these protein interactions as input and extracted the distinct proteins of each organism called seeds to build new candidate PPIs. We correlated the seeds to identifier names by searching the value of *skos: prefLabel* property in the semantic file exported by UniProt. Then we used the name of the seeds to retrieve their interaction partners in the STRING API. The number of retrieved bacteria PPIs was under 200, whereas the number of PPIs for the human seeds was 5142. *E.coli* had 146 PPIs, representing the highest number among all the bacteria species studied here. As the final results predicted by PredPrIn, the positively classified PPIs followed a similar proportion in relation to the input, ranging from 87 in *P.aeruginosa* to 6187 in *E.coli*.

**Table 1.** The first row corresponds to all validated PPIs between each bacterial species and host protein in the HPIDB database

Dataset	*E.coli*	*M.tuberculosis*	*P.aeruginosa*	*S.aureus*
HPIDB PPIs containing any host protein	277	22	31	10 155
HPIDB PPIs only with human host as partner	191	15	18	39
String high confidence PPIs	146	48	1	4
New candidates for PredPrIn prediction	23 0547	66 933	2479	9916
Positively predicted PPIs	6187	4207	87	116
Hub analysis *P*-values	0.08	0.073	0.47	0.93

*Note*: The second row is a subset of the original HPIDB datasets containing only human proteins. The third row contains STRING PPIs (score of 900). The fourth row corresponds to all the candidate PPIs generated by combining the interaction partners in the neighborhood of the known host–pathogen PPIs. The fifth row is the final list of positively predicted PPIs by PredPrIn. The last row corresponds to the *P*-value found for each specie in the hub bias analysis.

We then asked if there would be a bias in PPI predictions due to the existence of the seeds from the pathogen and host with many interaction partners, called hub proteins (Vallabhajosyula *et al.*, 2009; Vidal *et al.*, 2011). To answer this question, we investigated whether there would be a deviation between host and pathogen seed degrees in the list of candidate PPIs (PredPrin input) and their respective degree. This was tested after predicting protein interactions with PredPrIn for each bacterial species. The statistical test of this hypothesis was executed by the *scipy.stats.pearsonr* (Pearson method implementation) function in the Scipy package (Virtanen *et al.*, 2020), in which the chosen alternative hypothesis was two-sided (correlation is non-zero). The results show that the frequency of predictions related to a particular seed was not significantly (threshold *P*-value ≤ 0.05) correlated with the degree of that seed, demonstrating that the model predictions are unaffected by the seed degree centralities (Table 1 and Supplementary Table S1).

#### 3.1.1 Comparison among PPIVPro and STRING text-mining scores

These PPIs for each organism were retrieved from STRING and used in the transitivity analysis (Figure 2). In this context, we studied the possibility of transferring the knowledge from validated PPIs to their interaction partners. Our goal was to analyze whether the partners of bacterial proteins of high confidence interact with host protein partners. Using these sub-PPI networks, we obtained the new PPI candidates by combining all partners of bacteria seeds with the partners of host seeds. As expected, *E.coli* and *M.tuberculosis* generated the highest candidate PPIs since they have the most seed quantities.

As OntoPPI and PPIntegrator enable the integration of supporting article information for the protein interactions, we evaluated the STRING interactions and the new positively predicted host–pathogen PPIs with our previously developed PPIVPro validation process (Martins *et al.*, 2021). While the HPIDB already provides the publications attached to each validated known host–pathogen PPI, the PPIntegrator preparation step links the corresponding literature information. In the step of interactions extracted from the STRING, we also obtained the scores provided by the STRING API and the protein pairs (from the text mining of the literature). Thus, we could compare the findings of these scores using the PPIVPro, as shown in Table 2. Although the criteria adopted by STRING to derive the text mining score are less sensitive (such as the mere presence of both proteins in a given sentence), our method in PPIVPro takes into account the sentence construction and interaction context rules. Besides, after these computationally inferred PPIs enter into the STRING database, they are not updated frequently with recent articles from the Pubmed database. In this way, the disagreements between these scores were high in the two most extensive datasets.

**Table 2.** Summary of text-mining-based scores for the STRING PPIs of each species

Agreement of scores	*E.coli*	*M.tuberculosis*	*P.aeruginosa*	*S.aureus*
First case—both high	11	2	0	0
Second case—PPIVPro high	66	13	1	2
Third case—TM-score high	24	18	0	0
Fourth case—both low	45	15	0	2

*Note*: The first case shows the number of PPIs whose text-mining STRING score (TM-score) was high, and PPIVPro also found sentences in articles mentioning the proteins in an interaction context. The second case shows the number of PPIs to which PPIVPro found sentences in articles, but the text-mining score was low. The third case occurs the opposite; the PPIVPro found no sentences correlating the proteins to interactions, but the TM-score was high. Finally, in the last case, both PPIVPro and TM-score were low.

The PPIVPro also evaluated the new positively predicted host–pathogen PPIs. The results show that 1816 PPIs have matches in Pubmed central papers mentioning them for *E.coli*. Still, only 64 PPIs passed the context filter implemented by the validation process method. The same behavior occurred for *M.tuberculosis*, in which 946 PPIs have matches in articles, but only 15 were related to interaction. PPIVPro provides a filtered set of sentences and articles with the two proteins in an interaction context. The PPIVPro results were refined by hand curation to ensure the quality of the findings.

Recent literature presents methods to augment PPI networks. Although some methods (Liu and Rajapakse, 2019) use mathematical models on graph walks to generate potential new protein interactions, our proposed method to discover new host–pathogen interactions uses a strict direct neighborhood of the protein seeds belonging to the known PPIs. We reduced the complexity of candidate generation and increased the PPI evaluation confidence by combining a recent PPI prediction method evaluated in several organisms’ datasets. The PPIVPro validation process then validates the predicted PPIs to discover evidence in the published literature to increase the confidence of the final potential host–pathogen PPIs. Most published methods use known information of validated protein interactions to enrich and generate new potential PPIs, mainly using the shortest paths across known validated PPIs pairs (Liu and Rajapakse, 2019; Nourani *et al.*, 2015). Similar to Kshirsagar *et al.* (2013), we combine all the validated PPIs available for the four bacteria studied in our paper using knowledge transfer to improve the model generalization. We handle the non-interacting data samples issue by modeling the training data with equal numbers of PPIs in a low score from STRING for these bacteria and humans. In this way, we ensure that the negative samples have no potential true host–pathogen PPIs.

In discovering new host–pathogen interactions, currently available methods can perform some tasks, such as augmentation protein interactions by generating new candidates, prediction process and post-processing analysis, but not all together. Specifically, the works addressing PPI augmentation generally use ortholog information to derive new potential PPIs for pathogens not registered in host–pathogen databases (e.g. Lee *et al.*, 2008; Loaiza *et al.*, 2021). These methods are advantageous because they form new interactions by propagating protein interactions from orthologs in other pathogen networks. Nevertheless, our method may contour this disadvantage of requiring validated PPIs as seeds using the homolog search option from HPIDB database to search validated interaction seeds to start our proposed workflow. Regarding the host–pathogen protein interactions prediction, some methods use primary sequence to generate features with additional approaches, such as functional annotation and domain–domain interactions submitted to classifiers (Zhang and Skolnick, 2005), multi-model consensus (Zheng *et al.*, 2021) or simple filtering (Huo *et al.*, 2015). Despite these approaches utilizing diversified biological information, most of them are developed for specific organisms [such as the fungus *Magnaporthe oryzae* (Zheng *et al.*, 2021), and *Mycobacterium tuberculosis* (Huo *et al.*, 2015). Besides, they also do not allow the generation of new PPI candidates. In this context, we proposed a scientific workflow integrating the PPI augmentation process to generate new PPI candidates and perform PPI prediction and post-processing analysis tasks. Our infrastructure workflow may be used for any host–pathogen data, is scalable, enables parallel execution of multiple datasets, and is well documented to make it reproducible.

In this work, we proposed a method to predict new host–pathogen protein interactions and expand the PPI network. Our method allows multiple host–pathogen protein interaction predictions in parallel that scale according to the machine resources since it was built as a reproducible workflow infrastructure. We chose to predict them with a method that allows full interpretability by using biological-based inference methods to support the final classification. Recent strategies have been used to take advantage of the graph natural interpretation of the protein interactions such as Bayesian Networks (Deeter *et al.*, 2017) and Graph Neural Networks (Zhou *et al.*, 2022). The former relies on conditional probabilities that are learned to generate new probabilities for edges among proteins, and the latter generates embedding representations based on graphs and kernel functions to generate feature vectors that will be classified. In both cases, the results are promising, but they need to be restructured to include the biological background for the predictions.

### 3.2 Semantic integration of PPI networks

Once finished with the enrichment of the host–pathogen PPI network, the PPIs retrieved from HPIDB, STRING and the new predicted PPIs were triplified using our proposed method PPIntegrator. The tool to store the semantic graph of protein interactions was the AllegroGraph, which allows adding the inferred triples in the query.

The semantic data integration of all networks enables the global analysis of interactions from heterogeneous databases. Then, we explored the enriched functional annotations for each GO category to assess the most common functions for interaction pairs containing Humans as the host. We compared the functional enrichment for the HPIDB and PredPPI positively predicted interactions through SPARQL queries on the semantic database. The annotations of the three categories (cellular component, molecular function and biological process) were grouped by dataset name, the UniProt accessions and the taxonomy id, and then filtered by the PredPrIn and HPIDB in the dataset names.

Then we evaluated the frequency and statistical significance (https://github.com/tanghaibao/goatools) of the functional enrichment according to the term hierarchy of the GO. The top-ranked were selected for each organism, ignoring the strain specificity. In the *E.coli* results, the protein phosphorylation was present in both ranked lists. We found another intersection for *S.aureus* concerning the cell adhesion biological process. The HPIDB had no representative annotations in this category for *M.tuberculosis* or *P.aeruginosa*. Whereas by the PredPrIn database, we found some processes for *M.tuberculosis*, such as carbohydrate metabolic and cell wall organization. However, we still found no term for *P.aeruginosa*.

According to the ranked terms in the cellular component category, the two databases (HPIDB and PredPrIn) obtained more intersections for the extracellular region, an integral component of the membrane and plasma membrane. Both ranked lists also agreed on the extracellular region for *S.aureus*. While the integral component of the membrane was the only subcellular location found by the PredPrIn, HPIDB ranked the extracellular region for *P.aeruginosa*. There was no hit for *M.tuberculosis* in the HPIDB ranked list, but PredPrIn also ranked this integral component of the membrane and cytoplasm.

Interestingly, many annotation terms ranked by both databases were related to metal binding in the molecular function category. Indeed, many host–pathogen interactions for bacteria need co-factors for catalytic activity (Cerasi *et al.*, 2013; Khan *et al.*, 2020; Shah *et al.*, 2015). In the *E.coli* list of the PredPrIn interactions, there was also great participation of terms related to kinase activities. While the GTPase activator activity function was in the top position in the HPIDB for *P.aeruginosa*, the chaperone binding was the top-ranked term in the PredPrIn interactions set. There was no term in the HPIDB for *M.tuberculosis*, but in PredPrIn ranked terms list, we found similar functions such as kinase activities, DNA and ATP binding, and metal binding.

The data organization model implemented by the PPIntegrator allowed the holistic and comparative analysis of all bacterial datasets. Our tool used specific ontologies projected for the protein interactions domain to perform the triplification, bringing together related annotation databases. Our tool deals with other identifiers in the data preparation step. As an advantage, PPIntegrator links the UniProt identifier for every case and allows the integration with other databases via the cross-references to external knowledge bases information stored in the UniProt. Although Arzt *et al.* (2011) did not use semantic web technologies, they integrated protein interaction data with pathways found in the KEGG. They merged these data with some protein functional features loading from UniProt and storing that in a local database. We designed PPIntegrator to export data in RDF, KEGG, UniProt and SPARQL queries, allowing us to run them on other graphs by federated queries. Thus, we do not need to make data redundant by bringing them to local storage.

Another improvement is that we created the PPIntegrator to allow the registry of experiment and datasets details. In similar regard, Kazemzadeh *et al.* (2014) developed a framework for PPI integration data, in which they created a vocabulary that includes a few classes, such as experiment, interaction (with the score attribute) and features information. However, this vocabulary lacks some vital information related to the PPI entire process concerning protein information acquisition, feature extraction, prediction and validation. For example, the description of the dataset information and the evidence method applied, in general, are not available.

Furthermore, PPIntegrator provides a manner to express biological information used for each pair component and associates the knowledge of what evidence method generated the interaction scores. In Cannataro *et al.* (2010), the authors enriched a database of PPI with GO terms. They also built a framework where the user can navigate through data using remote SPARQL queries, providing a public website to retrieve the information for topological analysis in the network. However, their disadvantage is that they lack score discrimination by evidence method and do not provide data about the provenance of the predicted PPI network. In a similar context, Dhanapalan and Chen (2007) do not explore other information about the detection methods and the functional annotations of the proteins. While the PPIntegrator uses specific ontologies projected for the protein interactions domain to do the triplification.

Besides, PPIntegrator facilitates the analysis and network filtering according to different aspects because it offers data exportation automatically for allegrograph allowing running queries on the semantic database created with PPIntegrator with reasoning inference. Moreover, PPIntegrator integrates databases containing PPIs with pairs of proteins from organisms of different species, such as host–pathogen interactions.

## 4 Conclusion

Here, we proposed the PPIntegrator system to automatically triplify and integrate protein interaction datasets from heterogeneous data sources and applied it to integrate PPI data from four pathogenic bacteria species. We also introduced the host–pathogen discovery process that combines graph-based candidate generation and two layers of evaluation for the prediction and validation of the new PPIs. The proposed host–pathogen discovery process can find selected candidates for posterior wet lab validation aside from those with relevant evidence in recent literature by our validation process (Martins *et al.*, 2021). We provide methods to deal with all the processes, from generating new potential PPIs, passing through the prediction, and then post-processing analysis. Our workflow infrastructure also allows parallel execution of multiple datasets and execution monitoring, and it can be used for any host–pathogen dataset. We showed the holistic functional analysis comparing the PPI networks before and after the augmentation showing the gain of information and the new knowledge aggregated by our two strategies. The comparative functional analysis of the bacterial protein interactions network showed the biological similarity given by the shared molecular functions among the known and new host–pathogen interactions using the integrated semantic database, indicating the robustness of our strategy. Although PPIntegrator was used in host–pathogen PPI data, the system is flexible and accepts protein interaction data of any organism preparing data from several source databases.

## Supplementary Material

vbad067_Supplementary_Data

## Data Availability

The data underlying this article are available in the article and in its online supplementary material. Additionally, the source codes of the methods are found in: https://github.com/YasCoMa/ppintegrator, https://github.com/YasCoMa/ppi_validation_process and https://github.com/YasCoMa/predprin.
