# Supplementary material for: PPIntegrator: semantic integrative system for protein–protein interaction and application for host–pathogen datasets
Source: Bioinform Adv. 2023 Jun 1;3(1):vbad067. doi: 10.1093/bioadv/vbad067 (PMC10290227; doi:10.1093/bioadv/vbad067)
Supplement: vbad067_Supplementary_Data [file vbad067_supplementary_data.zip › supplementary_material.pdf]

Supplementary material corresponding to the article “PPIntegrator: Semantic integrative system for protein-protein interaction and application for Host-Pathogen datasets”

Section S1 refers to the concepts of vocabularies, ontologies, dataset descriptions using the Resource description framework and details of the namespaces used across the article. Section S2 describes the OntoPPI ontology in detail and illustrates the classes as well as the data and object properties. In section S3, we describe the data extraction and transformation processes for each of the databases that PPIntegrator accepts as input. Finally, S4 describes and illustrates the Triplification and Data Fusion Module of the PPIntegrator.

Supplementary Material S1. Background concepts on semantic web

The semantic web represents an evolution of the way that data is published on the web so that not only humans can understand, interpret and perform reasoning but also the machine can capture the meaning of the concepts and their relationship (Bizer, Heath, and Berners-Lee 2011). To achieve this goal, the data is organized according to the specifications of the Resource Description Framework (RDF), in which a certain knowledge area domain is described according to triples. Each triple is composed of three elements: subject, predicate and object. The subjects are resources that may be classes or instances of classes, the predicate indicates the relationship between subject and object, and the object may be a Literal value (string, integer, float, etc) or a resource. These predicates correspond to properties that may be a data property when it connects a subject resource to a Literal, or an object property when it connects the subject to another resource. In order to describe the concepts of this domain, it is necessary to use classes and properties from some ontology or vocabulary (Candan, Liu, and Suvarna 2001).

The ontologies and vocabularies specify the classes, the properties and the constraint rules. These properties may have descriptions that help automatic reasoning leading to new inferred triples such as those descriptions defining their domain (subjects allowed to use it) and range (type of the target object) (Garijo and Poveda-Villalón 2020; Hartmann et al. 2005). All the resources and items described in datasets, ontologies and vocabularies have a unique uniform resource identifier (URI) in order to be accessible, findable and interoperable (Boeckhout, Zielhuis, and Bredenoord 2018). These URIs are formed using Namespaces, which are represented by short aliases used to abbreviate the canonical source of the objects, classes or properties of a certain vocabulary or ontology. Below, there is a table with the description of the main namespaces used along this article.

Name	URL
geo	http://rdf.geospecies.org/ont/geospecies#

biomanta	http://biomanta.sourceforge.net/2007/07/biomanta_extension_02.owl#
biopax	http://www.biopax.org/release/biopax-level3.owl#l
uniprot	http://purl.uniprot.org/core/
owl	http://www.w3.org/2002/07/owl#
rdfs	http://www.w3.org/2000/01/rdf-schema#
uniprotTaxon	http://purl.uniprot.org/taxonomy/
**ontoppi**	**https://www.ypublish.info/protein_interaction_domain_ontology#**

Ontologies have been used in many knowledge areas. Along the last years many ontologies emerged and have been broadly used in a wide range of applications. One case of application is in the biology field, in which many concepts overlaps with each other and there is a need for data exchange and interoperability (Whetzel et al. 2011; Konopka 2015). The most known case is the Gene Ontology (GO) that describes functional annotations of Genes such as molecular function, cellular component and biological process (Gene Ontology Consortium 2015). The Uniprot database (UniProt Consortium 2019) that encapsulates information about protein sequence, functional annotation, structures and external database references also has its own vocabulary and provides external mapping among the proteins it represents and their respective GO terms. These semantic descriptions (mappings) allow the interoperability and aggregation of knowledge graphs to enrich a dataset and perform advanced queries to analyze data.

Supplementary Material S2. OntoPPI

OntoPPI is an ontology designed to describe the concepts involved in a computational experiment of protein interactions prediction. It allows the representation of the datasets content and provenance, the proteins functional annotations, the prediction methods and the results of the prediction. Figures 1 and 2 show the OntoPPI classes with their respective data and object properties.

OntoPPI includes the ontoppi:Experiment class, which is a subclass of the biomanta:PredictObservation class. This association allows the experiments being inferred in biomanta ontology as *in silico* prediction experiments and use specific properties in case of the dataset being described be derived from computational observations. The Experiment class is associated (ontoppi:usesEvidenceMethod) with the ontoppi:EvidenceMethod class, which represents the methods that can be used for an experiment. However, it is important to highlight that the Experiment class aims to represent computational and wet experiments. In addition, the Experiment class is associated (ontoppi:hasDataset) with the ontoppi:PPIDataset class, representing datasets used in both kinds of experiments.

A typical PPI dataset contains a set of candidate protein pairs. Each protein in OntoPPI is represented in the ontoppi:PairComponent class, which is a subclass of biopax:Entity and corresponds to (ontoppi:hasUniprotCorrespondent) a protein identifier from Uniprot database[1]. To represent the candidate protein pair, we reused the biopax:Interaction class,

1 https://uniprot.org/

which is associated with two components (ontoppi:PairComponent). Each candidate pair is associated with a PPI dataset. This association is represented by the ontoppi:belongsTo property.

Finally, biopax:Interaction is also associated with ontoppi:hasScore and with ontoppi:EvidenceDecision class to represent the results of evidence methods. Additionally, ontoPPI:EvidenceDecision class is associated (ontoppi:basedOn) with the ontoppi:EvidenceMethod class, to represent the method in which an evidence decision was based on.

Considering the experiment process and the concepts reused from the mentioned ontologies, OntoPPI data flow starts with the descriptors about experiment configuration. Experiment class, in this context, means a PPI experiment that can use one or a set of datasets to evaluate protein pair candidates.

The Experiment class properties are *hasOwner*, *hasContactEmail*, and *hasDescription*. The *hasOwner* property informs who is responsible for the data provided and for the experiment. The *hasContactEmail* property has the information about contact email that can be used to inform the status of experiment execution. The *hasDescription* property has as domain a collection of three classes, namely *EvidenceMethod*, *Experiment* and *PPIDataset*. This property can be used to give details about the experiment, such as goals. For the PPIDataset class, this property can be used to describe details of each dataset, like the number of pairs, the proportion of negative and positive ones, and how the datasets were formed (original database where the pairs came from (for instance, STRING[2])). Finally, this property can also be used to describe details of EvidenceMethod, such as how the method calculates its score from the biological information about the protein pairs.

The object properties of Experiment class are hasDataset and usesEvidence Method. The range of hasDataset is the PPIDataset class. The range of usesEvidenceMethod is the EvidenceMethod class. PPIDataset represents a PPI Dataset given for evaluation that normally contains pairs known to interact and others to train as false examples. Each line of the false and positive files contains two database identifiers, one for each protein. One example is the identifier from a database named Uniprot, which has information about the protein and annotations/linkage to other databases to enrich the information.

PPIDataset class has two datatype properties: (i) hasFolder property represents the address where preprocessing step can find the files mentioned before starting the experiment; and (ii) hasPrefix property represents a pattern to identify the false and positive file names with the pairs.

The EvidenceMethod class is used to represent the different methods to calculate and analyze the possible interactions between proteins. This class also has the hasType property, which indicates the kind of evidence method. The evidence methods can be classified into the following types: evolutionary relationship; functional features; network

---

2 https://string-db.org/

topology; sequence-based signatures; structure-based signatures; and text mining (Kotlyar et al. 2015).

The PairComponent class represents a protein involved in a protein pair, and has the following properties: hasGO_cc_annotation, hasGO_bp_annotation, hasGO_mf_annotation, hasPfam_annotation, hasKo_annotation, and hasUniprotCorrespondent (unique object property, the others are datatype properties). The hasGO_cc_annotation property can be used to annotate all GO terms in the cellular component branch that the protein has. The hasGO_bp_annotation property can be used to annotate all GO terms in the biological process branch. The hasGO_mf_annotation property can be used to annotate all GO terms in the molecular function branch assigned to the protein.

The hasPfam annotation property can be used to annotate all the Pfam Database[3] identifiers (i.e., protein families) assigned to the protein. The hasKo_annotation property can be used to annotate enzyme identifiers of metabolic pathways stored in the KEGG database[4]. The hasUniprotCorrespondent property links the pair component with their correspondent in the Uniprot database.

The range of this property is the class Protein, found in the Uniprot ontology. The Interaction class from Biopax was reused as its definition is the same for our domain and we add some properties to it. Two properties were adapted from an already existent property in Biopax for this class, i.e. participant. The two properties added were participant1 and participant2, while their range is the PairComponent class. The goal of this change is to improve the efficiency of query results because an interaction always involves two components. The results using only one participant implied a duplication for the results of interaction scores because the second participant was not explicitly described, which was solved by defining those two properties. The Interaction class also has the following properties: belongsTo and hasScore. The belongsTo property is a link between the interaction and the PPI dataset where it came from, so its range is the PPIDataset class. The hasScore property has as range the EvidenceDecision class and links the interaction with the probabilities generated by the evidence methods.

The EvidenceDecision class allows establishing the relationship between the interaction and all score results that it has according to each evidence method. The EvidenceDecision class has the object property basedOn and the data property predictedValue. The basedOn property connects an instance of the EvidenceDecision class to an instance of the class EvidenceMethod, and links to the evidence method that generated the corresponding score. Finally, the predictedValue property can be used to attribute the score value (between 0 and 1).

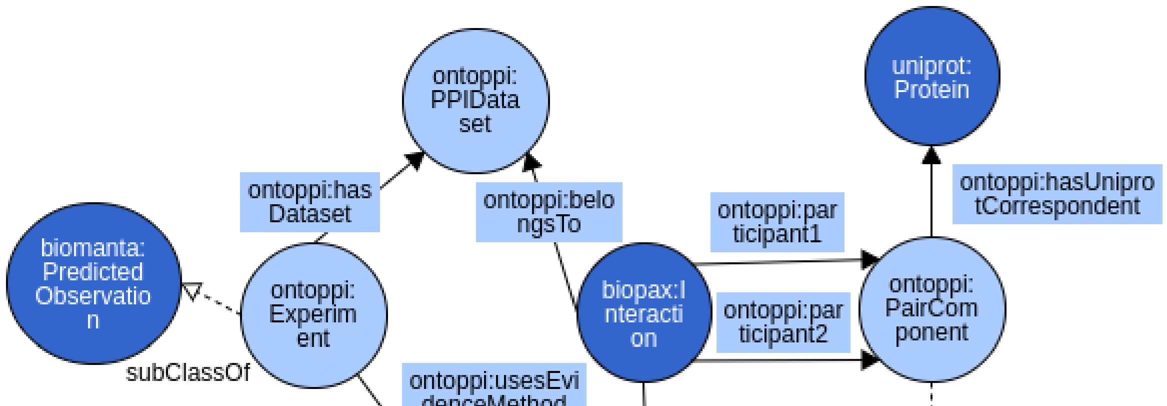

Figure 1. OntoPPI classes and object properties. Light blue circles represent OntoPPI classes while the dark blue ones represent classes reused from external vocabularies. The light blue rectangles mean the object properties, whose range corresponds to a class.

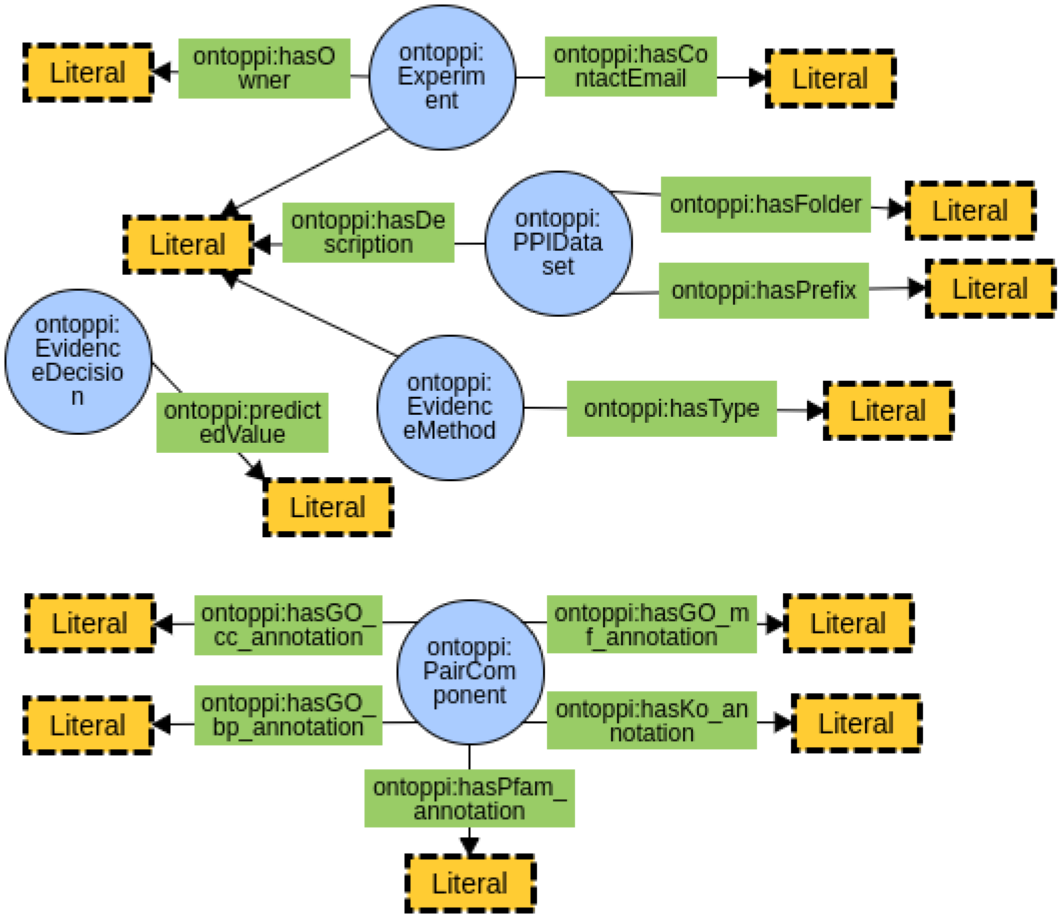

Figure 2. OntoPPI classes with their respective data properties. Light blue circles represent OntoPPI classes, green rectangles illustrate the data properties whose range may be any standard values such as strings, numbers. These values are generalized by the name Literal, shown in the figure by the yellow rectangle.

Supplementary Material S3. Details of PPIntegrator data transformation

The data preparation requires a file containing the experiment configuration. This file has the provenance details of the experiment that originated the datasets with interactions. The information related to each experiment is the name, description, the owner (person or group responsible for the datasets), the owner's contact email, and the dataset information. The information of the datasets is the organism of its interactions (using the complete scientific name to avoid any variations), the name, description, the prefix used to name the positive and false files with the protein pairs, and the folder where the files of the experiments are stored. All this information is organized in a json file.

The information described above has to be personalized for each database, and this file and its fields have the purpose to organize data for every experiment regarding protein interactions. Each of these three data sources used for integration exports its information differently. Due to the heterogeneous files provided by these sources, we treated the data preparation for each case.

- PredPrIn case

This data source already provides information about the functional annotations of the proteins involved in the interactions. This information is used in step two of the triplification module. The other file is the dataset with the protein pairs, and it has at least two mandatory columns for the protein identifiers (Uniprot) in a tab-separated values (TSV) file. This source also had this dataset with a third extra column with the label of each pair. The last file needed was the complete dataset with each column representing the scores of the detection methods also used in TSV. PredPrIn exports the file with the scores separated by space, and the last column has the original label for the pair to which these scores belong. So, we rewrite this file, leaving just the numerical features with the score, and separate them with tabulation.

- STRING case

This data source does not provide the file with functional features of the proteins involved in the pairs. The triplification module does not require this file, instead it just helps to enrich and filter the data according to the protein characteristics. This dataset provides a lot of files concerning the interactions stored in it for further analysis. The file used by PPIntegrator is the one containing the protein interactions with the scores per channel (this channel refers to each detection method). As this file provides protein identifiers using the Ensembl identification, we use the mapping file, also available in STRING, to map this identifier to UniProt. So, the two first columns correspond to the two proteins involved in the PPIs. This provides the dataset file, and the subsequent columns of information are used to give the dataset of numerical features with the scores for each detection method. Using a dictionary, we mapped each Ensembl identifier to UniProt. The scores per channel file separate the columns using space, and the mapping file uses tabulation. These scores were normalized to fit and maintain the pattern of values between 0 to 1, and this database returns the scores between 0 to 1000 then we divide all the values by one thousand.

- HINT and HPIDB cases

These data sources do not provide the file with detailed protein information. HINT and HPIDB offer the download of binary relations between proteins. As these interactions are from literature curation or experimental validation, all these interactions are assumed to have a score of 1 (maximum). We defined this score as a general score. A portion of the interactions stored in these databases came from DIP (Database of Interacting Proteins), and this database offers full files for six organisms containing protein pairs, the name of the laboratory detection methods (not *in silico* methods), and the PubMed identifiers. These files contain the protein identifiers using the UniProt identifier and the one given by DIP, but we just maintained the UniProt one. This information formed a new TSV file with three columns that was another input for the next module. When this file exists, the module associates the evidence decision of the interaction with its respective PubMed[5] identifiers.

In these three cases, we generated the required files: the PPI dataset with the protein pairs, the dataset with numerical features, which are scores of each detection method, and the file with corresponding articles related to the pair of proteins. The output files of the preparation module are stored in the directory described in the configuration file received as input. This file is the same as the one used in the Triplification and Data fusion module of the PPIntegrator.

Supplementary Material S4. Example of dataset descriptions preparation

The PPIntegrator triplification process illustrated in Figure 3 starts by describing, in Step 1, the protein interaction experiment (*hppi* is the namespace of the example dataset) *ExperimentEx2*. Then, it links this experiment with the dataset *DatasetDs1*, which is located at the folder */home/user/ds1*, and the experiment owner is *Maria*. This dataset contains two proteins (*P9WIA1* and *Q8N5C8*), the first protein comes from the organism of taxonomic id 83332 that is the *Mycobacterium tuberculosis*. The latter is from the Human organism with taxonomy id 9606. The nodes concerning the taxonomic identifiers are resources from Uniprot Taxon vocabulary, linked to their scientific names by the Geospecies data property named *hasScientificName*. At this point, these proteins are included in the semantic graph but they are still dissociated from other elements, i.e., they are not connected to the interaction node.
Step 2 is responsible for adding the functional annotations to the protein nodes with the gene ontology, KEGG or Pfam identifiers. So, the protein *P9WIA1* in this example is linked to its molecular function identifier *GO:0004725* and the biological process identifier *GO:0052067*. The protein *Q8N5C8* is linked to the annotation of cellular component *GO:0010008* and the family annotation from Pfam database (*PF02845*).
Finally, the step 3 connects the proteins in the respective interaction (*Interactionv5gv6g*) that belongs to the *DatasetDs1*, that was predicted using the evidence decision *DecisionEv1* with the value 0.96 based on the method (*MethodEm1*), which is *PredPrIn*.

[5] https://pubmed.ncbi.nlm.nih.gov/

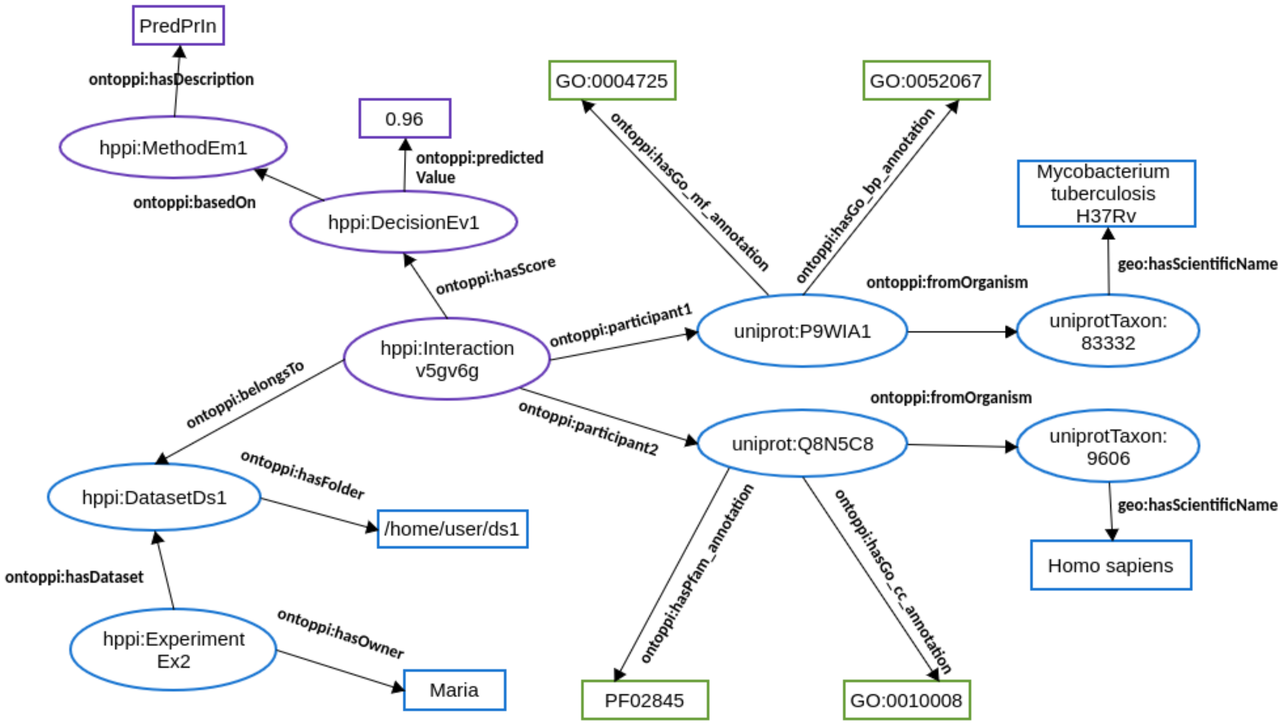

Figure 3. Example of semantic descriptions generated by PPIntegrator. The blue nodes are added by step 1 referring to organisms, dataset and experiment descriptions; the green nodes represent the functional annotations of the proteins inserted, when available, in step 2; finally, the purple nodes correspond to the methods and the results of the predictions for each protein interaction.

Bizer, Christian, Tom Heath, and Tim Berners-Lee. 2011. “Linked Data: The Story so Far.” In *Semantic Services, Interoperability and Web Applications: Emerging Concepts*, 205–27. IGI Global.

Boeckhout, Martin, Gerhard A. Zielhuis, and Annelien L. Bredenoord. 2018. “The FAIR Guiding Principles for Data Stewardship: Fair Enough?” *European Journal of Human Genetics: EJHG* 26 (7): 931–36.

Candan, K. Selçuk, Huan Liu, and Reshma Suvarna. 2001. “Resource Description Framework: Metadata and Its Applications.” *SIGKDD Explor. Newsl.* 3 (1): 6–19.

Garijo, Daniel, and María Poveda-Villalón. 2020. “Best Practices for Implementing FAIR Vocabularies and Ontologies on the Web.” *arXiv [cs.DL]*. arXiv. https://books.google.com/books?hl=pt-BR&lr=&id=gS4NEAAAQBAJ&oi=fnd&pg=PA39&dq=semantic+ontologies+and+vocabularies&ots=5Gy4zGj0vA&sig=m9XwCOzSusN4vT3wGxvW-5P-qHM.

Gene Ontology Consortium. 2015. “Gene Ontology Consortium: Going Forward.” *Nucleic Acids Research* 43 (Database issue): D1049–56.

Hartmann, Jens, Raúl Palma, York Sure, M. Carmen Suárez-Figueroa, Peter Haase, Asunción Gómez-Pérez, and Rudi Studer. 2005. “Ontology Metadata Vocabulary and Applications.” In *On the Move to Meaningful Internet Systems 2005: OTM 2005 Workshops*, 906–15. Springer Berlin Heidelberg.

Konopka, Bogumil M. 2015. “Biomedical ontologies—A Review.” *Biocybernetics and Biomedical Engineering* 35 (2): 75–86.

Kotlyar, Max, Chiara Pastrello, Flavia Pivetta, Alessandra Lo Sardo, Christian Cumbaa, Han Li, Taline Naranian, et al. 2015. “In Silico Prediction of Physical Protein Interactions and Characterization of Interactome Orphans.” *Nature Methods* 12 (1):

79–84.
UniProt Consortium. 2019. “UniProt: A Worldwide Hub of Protein Knowledge.” *Nucleic Acids Research* 47 (D1): D506–15.
Whetzel, Patricia L., Natalya F. Noy, Nigam H. Shah, Paul R. Alexander, Csongor Nyulas, Tania Tudorache, and Mark A. Musen. 2011. “BioPortal: Enhanced Functionality via New Web Services from the National Center for Biomedical Ontology to Access and Use Ontologies in Software Applications.” *Nucleic Acids Research* 39 (Web Server issue): W541–45.
